# Can non-invasively detected liver fibrosis using serum-based scores really improve risk stratification in patients with acute coronary syndrome?

**DOI:** 10.1007/s00392-023-02212-5

**Published:** 2023-04-28

**Authors:** Hans-Michael Steffen, Anna Martin, Sonja Lang, Philipp Kasper

**Affiliations:** 1 Clinic for Gastroenterology and Hepatology, University Hospital of Cologne, 50924 Cologne, Germany; 2 Hypertension Center, University of Cologne, Cologne, Germany

Sirs,

We have read, with great interest, the paper by Biccirè et al. [1] investigating the relationship between non-invasively detected liver fibrosis and in-hospital outcomes in patients with acute coronary syndrome (ACS) from a prospective real-world observational registry in Rome, Italy. However, we have second thoughts about the notion to use scores based on aminotransferase levels in this acute clinical situation.

Any longstanding hepatocellular damage can be accompanied by abnormal liver function tests and will lead to fibrosis and finally cirrhosis independent of the originally causing agent. Serum-based scoring systems, among others FIB-4 and APRI, were originally developed to detect advanced fibrosis in viral hepatitis with the intention to identify patients who would need antiviral therapy to prevent liver-related complications. These scoring systems have also been validated as screening tools for fibrosis in non-alcoholic fatty liver disease (NAFLD) and are endorsed by professional societies [2, 3]. The diagnostic accuracy can be improved with ultrasound- or MRI-based techniques assessing liver stiffness [4].

The proportion of patients with a history of chronic liver disease in the present study was very low at about 2.6%. The role of fibrosis as cardiovascular risk factor is controversial in cases of viral hepatitis, especially when direct antiviral agents are used [5, 6]. In addition, it is hard to believe that none of the included patients had known NAFLD, which represents the most common chronic liver disease worldwide and currently is estimated to affect about 25% of the adult European population.

NAFLD is considered as the hepatic component of the metabolic syndrome and is associated with an increased risk for the development of various liver-associated and cardiometabolic complications [7]. Most patients suffer from early stages of NAFLD with an estimated prevalence of advanced fibrosis or cirrhosis in 3.3–10% of cases [3, 8]. Compared with matched population controls, patients with biopsy-proven NAFLD had a significantly higher incidence of major adverse cardiovascular events (including ischemic heart disease, stroke, heart failure, and cardiovascular mortality), and the excess risk was evident across all stages of NAFLD [9].

The abovementioned scoring systems, namely FIB-4 and APRI, take levels of aspartate aminotransferase (AST/GOT) and alanine aminotransferase (ALT/GPT) into account and rely on the assumption that the diseased liver is the source of raised enzyme activities measured in the serum. However, this assumption is violated when the increase in serum levels is obviously related to cardiomyocyte damage as in case of ACS. Elevated serum levels of AST/GOT are often observed in patients with acute myocardial injury and a rough correlation between the height of the serum AST/GOT activity and the size of a myocardial infarction has long since been known [10]. It is, therefore, not surprising that patients with high AST/GOT levels because of severe myocardial cell damage have a higher rate of in-hospital adverse events accompanied by an increase in calculated FIB-4 values. It would be interesting to see, whether re-analyzing the data simply using serum AST/GOT activity alone would also identify the same patients with more severe ACS presentation and/or worse in-hospital adverse events.

In conclusion, we believe that the relationship between liver fibrosis and in-hospital outcomes in the clinical setting of ACS must rely on ultrasound- or MRI-based techniques assessing liver stiffness [4] to circumvent the problem of collinearity, inherent in scoring systems based on non-specific enzyme activities in the serum. The alternative could be to use AST/GOT values from visits prior to or after the index hospitalization. We are concerned that if these data are not available, conclusions of the assumed association are flawed.

## Data Availability

This must be a misunderstanding, there is no data availability statement necessary.
